# A report on the first conference on physiological and physical employment standards, Canberra, Australia November 27 to 28, 2012

**DOI:** 10.1186/2046-7648-2-14

**Published:** 2013-05-01

**Authors:** Gemma S Milligan, Michael J Tipton

**Affiliations:** 1 Extreme Environments Laboratory, Department of Sport and Exercise Science, Spinnaker Building, University of Portsmouth, Cambridge Road, Portsmouth, Hants, PO1 2ER, UK

**Keywords:** Physiological employment standards, Occupational tasks, Workloads, Load carriage, Injury

## Abstract

This is a report of the First Conference on Physiological and Physical Employment Standards. This was the first conference of its kind, attended by scientists, physicians, occupational medics, high-ranking politicians and military personal from ten nations.

## Scope of the meeting

The conference, held in late November in Canberra, was the first international research meeting to focus specifically on physical employment standards (PES). The focus was on the development, implementation and justification of PES within physically demanding civilian and military occupations. At the conference, leading scientists came together to present research related to the development of PES and to discuss areas such as the relationship between PES and the optimisation of workforce capability within contemporary and diverse societies, the difference between PES designed to be age- and sex- free or age and sex neutral, what constitutes a legally defensible PES, the role of subjectivity in the development of PES and differences in the philosophies and methodologies used to develop PES. Over the 2-day meeting, 42 oral presentations and 10 poster presentations took place. Thirty-five of these dealt specifically with the development and implementation of PES, the remainder introduced associated topics, such as load carriage performance and the problems and injuries associated with physically demanding jobs.

## Designing a physiological employment standard

The development of a valid and defensible PES has become important due to international legislation concerning age discrimination, coupled with requirements to ensure that employment selection is fair and unbiased, i.e. PES should be non-discriminatory. There were presentations on the PES developed for a number of occupations including the military (Sam Blacker, Optimal Performance, UK; Daryl Allard, Tara Reilly, Michael Spivock, Daniel Theoret, Director General Personnel and Family Support Services (DGPFSS) National Defence, Ottawa, Canada), rescue services (Gemma Milligan, University of Portsmouth, UK; Nigel Taylor, Alison Donohoe, Hugh Fullagar, and Herbert Groeller, University of Wollongong, Australia) and oil companies (Gemma Milligan, James House, Michael Tipton, University of Portsmouth). A number of groups presented work discussing the methodology undertaken to design a PES. In designing PES, all at the meeting agreed that the first stage is to perform a task analysis in which the physically demanding tasks of a job are identified. DGPFSS National Defence (Ottawa, Canada) presented a comprehensive methodological approach for task analysis/identification that should be considered when developing PES. There was some debate regarding the additional stages for the development of PES; however, the general consensus from several presentations was that to design a valid and defensible PES, the following criteria should be met:

1. Establish the physically demanding tasks considered to be critical to the safe and successful completion of a job through task analysis - determine the number and nature of tasks to be included.

2. Determine the Method of Best Practice for undertaking the critical tasks.

3. Agree on an acceptable minimum level of performance on the critical tasks.

4. Collect physical and physiological data: establish the demands associated with the critical tasks and decide upon the most appropriate descriptive statistical measure (e.g. the minimum, maximum, average, percentile, mode, median, etc.) to optimise employability, without sacrificing the ability to perform the critical task.

5. Determine a reasonable maximum permissible relative workload, e.g. the percentage of an individual’s maximum work capability; it is reasonable to expect them to work at or the time taken to complete a job demand circuit.

6. Production of a minimum occupational fitness standard.

a) Consideration of the use of task simulations or predictive tests, calculate and manage the imprecision (unexplained variability) of predictive tests.

a) Determine how to manage the false positives and false negatives that will be produced by predictive tests.

The consensus at the conference was that this methodology should ensure that PES are based solely on the physical requirements to undertake a job (task-related) and should therefore be independent from sex and age (Tipton, Milligan, Reilly, University of Portsmouth, UK; Yoram Epstein, Ran Yanovich, Daniel Moran, Yuval Heled, Tel Aviv University, Israel).

In addition, the conference addressed the need for PES to be legally defensible (Veronica Jamnik, Robert Gumieniak, Norm Gledhill, School of Kinesiology and Health Science, Toronto, Canada). For example, in Canada, the legislation states that for any PES to qualify as a *bona fide* occupational requirement and satisfy the Supreme Court of Canada, the following criteria must be met:

a. *That the employer adopted the standard for a purpose rationally connected to the performance of the job.*

b. *That the employer adopted the particular standard in an honest and good faith belief that it was necessary to the fulfilment of that legitimate work-related purpose.*

c. *That the standard is reasonably necessary to the accomplishment of that legitimate work-related purpose.*

It became apparent during the conference that a number of the critical tasks, identified by various presenters across a number of occupations, were similar in terms of physical and physiological requirements; these include tasks such as casualty drags, carrying and lifting loads, ladder climbing and stretcher carrying.

## Additional areas addressed

The remaining 17 presentations focused either on the problems and injuries associated with physically demanding jobs or load carriage. The majority of the presentations given in the area of load carriage came from a military perspective and reported that the absolute loads military personnel are expected to carry are increasing, with load carriage requirements reported from 20 to 70 kg, indicating significant demands both physically and physiologically.

Stephan Rudzki (Australian Defence Force) reported that low levels of aerobic fitness correlated strongly with increased injury rates, with women being twice as likely to experience injuries during military training compared to their male counterparts. Martin van der Linden (Corporate Health Group, Australia) concluded that pre-employment functional capacity evaluations have contributed significantly to reducing the frequency and cost of injuries within high-risk industries.

## Areas for future consideration

From the discussion of the papers presented, several aspects of the design and implementation of PES requiring clarification were noted. These included standardisation of terminology, e.g. terms used to refer to the tasks underpinning PES are variously referred to as criterion, generic and/or critical or essential; standardisation of methods for determining these critical tasks; determining an agreed method of best practice for developing PES; the application of PES to individuals working in extreme environments; the determination of acceptable scaling methods for metabolic demand across individuals; and the determination of maximum permissible workloads for various occupational tasks.

It is concluded that the First Conference on Physiological and Physical Employment Standards successfully and comprehensively addressed the major issues associated with PES and brought together those working internationally in this area. It is hoped that this meeting will act as a catalyst for future meetings and a coordinated approach for the completion of the experimental work required to improve the objectivity, standardisation and defensibility of PES.

## Invited reviews

As a result of the conference, four invited reviews are to be published based on the keynote presentations. These are as follows:

1. Tipton MJ, Milligan GS, Reilly TJ: Physiological employment standards I: occupational fitness standards: objectively subjective? *Eur J Appl Physiol* 2013, in press.

2. Jamnik V, Gumienak R, Gledhill N: Physiological employment standards II: developing and implementing employment standards for safety-related occupations. *Eur J Appl Physiol* 2013, in press.

3. Nindl, BC, Castellani JW, Warr BJ, Sharp MA, Henning PC, Spiering BA, Scofield DE: Physiological employment standards III: physiological challenges and consequences encountered during international military deployments. *Eur J Appl Physiol* 2013, in press.

4. Epstein Y, Yanovich R, Moran DS, Heled Y: Physiological employment standards IV: integration of women in combat units—physiological and medical considerations. *Eur J Appl Physiol* 2013, in press.

Those wishing to obtain further information in the area and specifically on the topics discussed at the conference are directed to the conference proceedings: Taylor NAS, Billing DC (Eds): *Physiological and Physical Employment Standards I*. Wollongong, Australia: University of Wollongong; 2012, pp. 1–109. ISBN: 978-1-74128-220-7.

## Abbreviations

PES: Physical employment standard; DGPFSS: Director General Personnel and Family Support Services.

## Competing interests

The authors declare that they have no competing interests.

## Authors’ contributions

Both authors wrote, read, and approved the final manuscript.

